# Surgical Resection of a Shamblin Type III Carotid Glomus Tumor Without Preoperative Embolization: A Case Report

**DOI:** 10.7759/cureus.87556

**Published:** 2025-07-08

**Authors:** Luis E Ocampo-Guzmán, Emmanuel M DelCampo-Madariaga, Karen Parra-Villanueva, Juan M Gómez-Rodríguez

**Affiliations:** 1 General Surgery, Hospital Regional Universitario de Colima, Colima, MEX; 2 General Surgery and Cardiothoracic Surgery, Hospital Regional Universitario de Colima, Colima, MEX

**Keywords:** carotid glomus, complex surgical resection, paranganglioma, preoperative embolization, shamblin type iii

## Abstract

Shamblin type III carotid glomus tumors represent a major surgical challenge due to their intimate relationship with neurovascular structures. We present the case of a 33-year-old male patient with a type III carotid glomus tumor who underwent surgical resection without preoperative embolization or vascular bypass. The postoperative course included lesion of the hypoglossal nerve as the only complication. Technical aspects, the controversial role of preoperative embolization, and new proposals for tumor classification that could optimize surgical planning in this group of patients are discussed.

## Introduction

Carotid glomus tumors are rare, hypervascular paragangliomas that originate in the carotid body. They represent 65% of head and neck paragangliomas, with an incidence of approximately 1:30,000 [1]. They are characterized by slow growth and generally benign behavior, although they can reach significant size and involve adjacent structures.

The most widely used classification is that of Shamblin, which divides carotid glomus tumors into three types according to their relationship with the carotid arteries: type I is easily dissected, type II partially surrounds the vessels, and type III is intimately attached to the internal and external carotid arteries, with significant anatomical deformity [2]. This classification has proven useful in predicting surgical complexity and risk of neurological complications.

Surgical resection is the treatment of choice, especially in large or symptomatic lesions. In type III tumors, preoperative embolization has been proposed as a strategy to reduce intraoperative bleeding and facilitate resection. However, the evidence on its actual usefulness is conflicting [3]. Recent meta-analyses suggest that, although it reduces bleeding and surgical time, it does not significantly reduce the incidence of neurological injury [4,5]. Its use should be individualized according to anatomical factors and the experience of the surgical team [6].

## Case presentation

A 33-year-old male patient, with no relevant medical history, consulted for a pulsatile, slowly growing, right cervical mass, with approximately three years of evolution. Due to the considerable increase of the tumor, he was evaluated by the otorhinolaryngology service, where it was decided to take a biopsy, reporting a carotid glomus, for which he was referred to vascular surgery. Subsequently, he began to experience pain, for which he went for a follow-up. Physical examination revealed a firm, well-defined, laterally mobile mass, with no signs of compression of adjacent structures.

**Figure 1:**
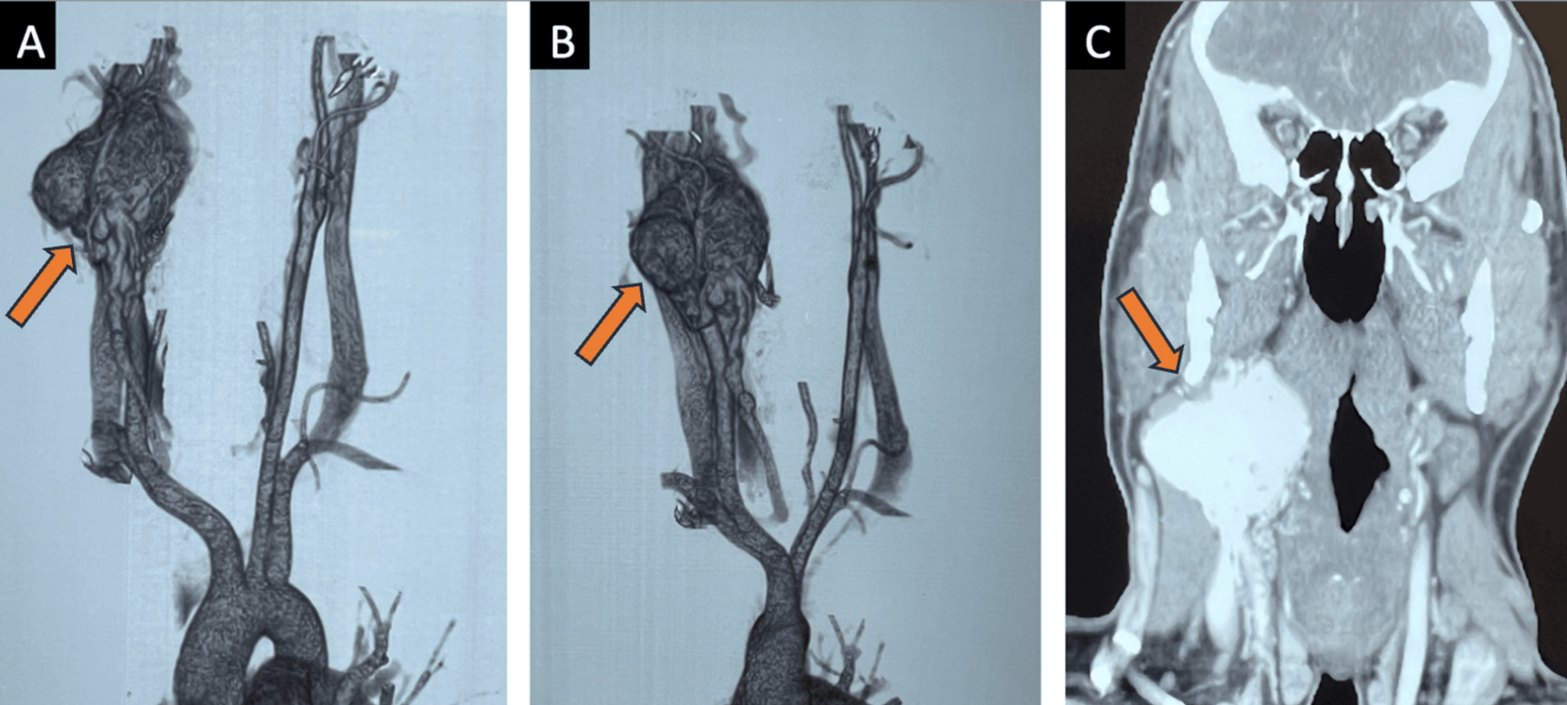
Angiotomography A-C show a hypervascular tumor at the bifurcation of the right carotid artery with lateral displacement of the internal and external carotid arteries.

Angiotomography showed a 5.2 cm hypervascular tumor in the right carotid bifurcation, with displacement of the internal and external carotid arteries laterally, showing medial growth without evidence of pharyngeal invasion (Figure 1A-1C). The tumor was classified as Shamblin III.

**Figure 2:**
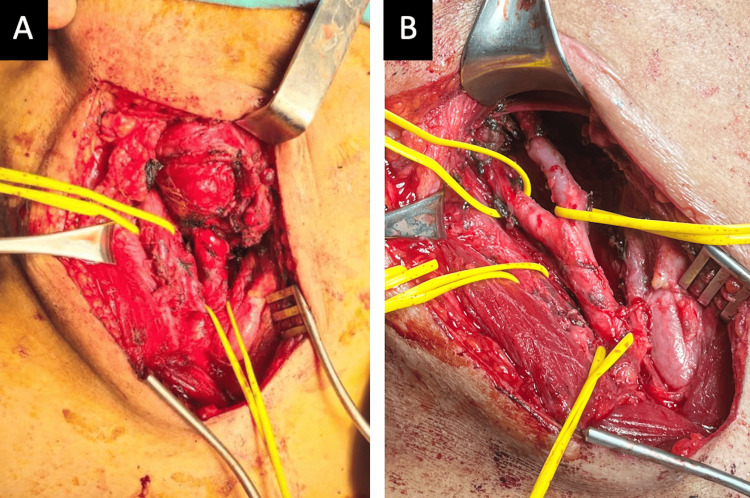
Carotid glomus tumor resection (A) Tumor involving common, internal, and external carotid arteries. (B) Carotid arteries can be seen after tumor resection.

After evaluation of the images, it was decided to proceed with surgical resection without preoperative embolization given the favorable access and to avoid the complications of embolization. During the procedure, extensive adhesion of the tumor to both carotid branches and the right hypoglossal nerve was evidenced. The resection was successfully completed with adequate vascular control (with careful dissection and early proximal and distal vascular control of the common, internal, and external carotid arteries by using vascular loops) and without the need for arterial reconstruction (Figure 2A-2B). The patient required one unit of packed red blood cells, administered intraoperatively, and the estimated blood loss during the procedure was approximately 1000 mL, mainly due to the tumor's high vascularity and close adherence to major vessels. Immediately postoperatively, the patient presented with ipsilateral lingual deviation, compatible with hypoglossal nerve neuropathy. No other neurological or vascular complications were observed. He was discharged on the fifth postoperative day, with outpatient rehabilitation and stable clinical follow-up.

## Discussion

Carotid glomus type III tumors represent the most complex variant from a surgical point of view due to their adhesion and displacement of vascular and nerve structures. The present case demonstrates that it is possible to perform a complete resection without embolization or arterial reconstruction, although with a minor neurological complication, that is, lesion of the hypoglossal nerve, one of the most frequent in this type of tumor [1,3].

The role of preoperative embolization continues to be a matter of debate. Several studies and meta-analyses conclude that, although it may decrease bleeding and operative time, it does not significantly reduce the rate of nerve injury or major vascular complications [2,4,5]. Moreover, embolization is not without risks, such as thromboembolic events, tissue necrosis, or distal vascular territory dysfunction [6,7]. Its usefulness may be more justified in large tumors, bilaterality, or institutions with limited surgical experience.

New classification proposals seek to improve surgical prediction beyond the Shamblin system. Ma et al. proposed an anatomic subdivision of carotid glomus type III tumors based on vascular relationships ("classical", "medial", "lateral", and "enveloped") and additional anatomic relationships ("common", "skull base", "pharynx", and "mixed"), allowing for more accurate planning according to the invasion pattern [8].

What makes this case particularly noteworthy is the combination of factors that depart from current common practice in the management of Shamblin type III carotid body tumors. First, the tumor was successfully resected without the use of preoperative embolization, a strategy often considered standard for large and hypervascular lesions of this type. Second, no vascular reconstruction was required despite the tumor's circumferential relationship with the carotid bifurcation. Lastly, although a hypoglossal nerve injury did occur, it was the only complication in a case that typically carries significant neurologic morbidity. These findings underscore that, in selected patients with favorable anatomy and experienced surgical teams, a tailored approach without embolization or bypass may be both feasible and safe.

## Conclusions

This clinical case reinforces the possibility of performing complete resections of Shamblin type III carotid glomus tumors without preoperative embolization or vascular reconstruction, provided there is surgical experience as well as adequate planning. Hypoglossal nerve injury remains a frequent complication, and its prevention depends on meticulous dissection. The incorporation of new anatomical-radiological classifications allows a more individualized and precise approach.
